# Autophagy and lysosomal dysfunction as emerging mechanisms of nanomaterial toxicity

**DOI:** 10.1186/1743-8977-9-20

**Published:** 2012-06-14

**Authors:** Stephan T Stern, Pavan P Adiseshaiah, Rachael M Crist

**Affiliations:** 1 Nanotechnology Characterization Laboratory, Advanced Technology Program, SAIC-Frederick, Inc., NCI-Frederick, Frederick, MD, 21702, USA

**Keywords:** Lysosome, Endocytosis, Autophagy, Nanomaterials

## Abstract

The study of the potential risks associated with the manufacture, use, and disposal of nanoscale materials, and their mechanisms of toxicity, is important for the continued advancement of nanotechnology. Currently, the most widely accepted paradigms of nanomaterial toxicity are oxidative stress and inflammation, but the underlying mechanisms are poorly defined. This review will highlight the significance of autophagy and lysosomal dysfunction as emerging mechanisms of nanomaterial toxicity. Most endocytic routes of nanomaterial cell uptake converge upon the lysosome, making the lysosomal compartment the most common intracellular site of nanoparticle sequestration and degradation. In addition to the endo-lysosomal pathway, recent evidence suggests that some nanomaterials can also induce autophagy. Among the many physiological functions, the lysosome, by way of the autophagy (macroautophagy) pathway, degrades intracellular pathogens, and damaged organelles and proteins. Thus, autophagy induction by nanoparticles may be an attempt to degrade what is perceived by the cell as foreign or aberrant. While the autophagy and endo-lysosomal pathways have the potential to influence the disposition of nanomaterials, there is also a growing body of literature suggesting that biopersistent nanomaterials can, in turn, negatively impact these pathways. Indeed, there is ample evidence that biopersistent nanomaterials can cause autophagy and lysosomal dysfunctions resulting in toxicological consequences.

## **Introduction**

Nanotechnology is an ever evolving field focused on the study of nanoscale materials. Recent applications of nanotechnology include material science, medicine and electronics. Concurrent with research into the application of nanotechnology is the study of potential risks associated with the manufacture, use, and disposal of nanoscale materials, and mechanisms of their toxicity. In order for the continued advancement of nanotechnology and its commercialization, it is important that these associated risks be identified and mediated. Currently, the most widely accepted paradigms of nanomaterial toxicity are oxidative stress and inflammation, but the underlying mechanisms are poorly defined [1]. This review will highlight the significance of autophagy and lysosomal dysfunction as emerging mechanisms of nanomaterial toxicity.

Endocytosis of nanomaterials by both phagocytic and non-phagocytic mechanisms most often culminates with lysosome internalization. The acidic pH and variety of hydrolytic enzymes (e.g., esterases, proteases, phosphatases, nucleases, and lipases) found in the lysosome represent an extremely hostile environment which can degrade all but the most biopersistent of these nanomaterials. In addition to the endo-lysosomal pathway, recent evidence suggests nanomaterials can also induce autophagy [2]. Among the many physiological functions, the lysosome, by way of the autophagy (macroautophagy) pathway, degrades intracellular pathogens, damaged organelles and long-lived proteins [3]. Thus, autophagy induction by nanomaterials may be an attempt to degrade what is perceived by the cell as foreign or aberrant. While the autophagy and endo-lysosomal pathways have the potential to influence the disposition of nanomaterials, there is also a growing body of literature suggesting that biopersistent nanomaterials can, in turn, negatively impact these pathways [2]. Indeed, lysosomotropic agents, including particles, have been known to cause lysosomal dysfunction and associated toxicity for several decades [4].

### **Endo-lysosomal pathways**

There are several potential pathways that can be involved in nanomaterial cellular uptake. Physicochemical properties of nanoparticles such as size, shape, and surface (e.g., charge and coating), as well as the cell type, all play a significant role in determining the predominant uptake pathway(s). For a more detailed discussion of the pathways utilized by specific nanomaterials, and commonalities with regard to cell types and nanomaterial characteristics, the reader is directed to several excellent reviews on the subject [5-7]. For review of imaging, inhibitor and quantitative methods used in delineating nanomaterial endocytic pathways, and issues associated with interpretation of these experimental methods, the reader is directed to the following literature [8-11].

**Figure 1:**
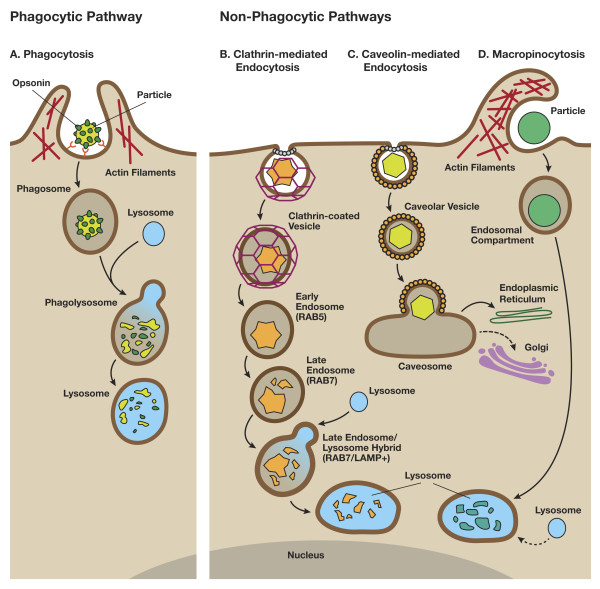
**An overview of phagocytic and non-phagocytic pathways. A)** Phagocytosis occurs in macrophages through an actin-based mechanism involving interaction with various specialized cell surface receptors (e.g., mannose, IgG, complement, Fcγ receptors). The foreign particles recognized by specific receptors, often targeting surface-bound opsonins, are internalized to form endocytic vesicles called phagosomes. The fusion of phagosomes with the lysosomal compartment leads to the formation of phagolysosomes, where the foreign particles are enzymatically degraded. **B)** Clathrin-mediated endocytosis involves the formation of vesicles from triskelion clathrin-coated regions of the plasma membrane. The triskelion clathrin in the cytosol are then recycled back to the plasma membrane followed by movement of ingested materials from early endosome to the late endosome, finally fusing with lysosome to form the lysosome-endosome hybrid. The materials are then degraded by the low pH and enzyme-rich environment of the endo-lysosomal vesicle. **C)** Caveolin-mediated endocytosis involves internalization through caveolin (a dimeric protein) enriched invaginations. The cytosolic caveolin vesicle then delivers its contents to endosomes, to form caveosomes which can avoid lysosomal enzymatic degradation, and are transported along the cytoskeleton to the endoplasmic reticulum/golgi complex. **D)** Macropinocytosis is a clathrin- and caveolin-independent pathway. It involves the formation of large vesicles called macropinosomes, which occurs through actin filament driven plasma membrane protrusions. The contents are degraded following fusion with the lysosomal compartment. (Figure partially adapted from Hillaireau and Couvreur, Cell. Mol. Life Sci. 2009, 66, 2873–2896).

Phagocytosis is an energy- and receptor-dependent uptake process involving invagination of the plasma membrane and subsequent fusion with lysosomes [12]. A cartoon depicting particle internalization by phagocytic and non-phagocytic pathways is shown in Figure1. In the phagocytic process of cellular uptake, actin filaments play a critical role, and inhibition of actin polymerization by cytochalasin D disrupts this internalization process. Phagocytic vesicles containing the ingested material fuse with the lysosomes to form phagolysosomes. The contents of the phagolysosomes are then degraded by enzyme-catalyzed hydrolysis. Phagocytosis takes place in specialized cells such as macrophages, monocytes and neutrophils, and functions in removing foreign particles [13]. The phagocytic pathway can utilize the binding of serum proteins (opsonins) to foreign particles, such as pathogens and nanomaterials, to aid recognition by specific macrophage receptors in a process termed opsonization [13]. Surface modification of nanomaterials with hydrophilic polymers such as polyethylene glycol, polyethylene oxide, and poloxamers can significantly reduce the opsonization of nanoparticles by serum proteins [14,15]. Avoiding recognition by the phagocytic cells of the mononuclear phagocytic system (MPS) (mainly liver and spleen) through surface coating can increase nanomedicine circulation, target cell exposure, and efficacy.

Non-phagocytic endocytosis (pinocytosis) can occur in most cells by several recognized mechanisms – clathrin-mediated endocytosis, caveolin-mediated endocytosis, clathrin- and caveolin-independent mechanisms, and actin-dependent macropinocytosis [12]. Several endocytic processes may be operative simultaneously in a cell, and the mechanism of uptake of a nanoparticle may vary within and between different cell types [5-7]. Clathrin mediated endocytosis (CME) is commonly involved in the transport of macromolecules, and receptor-dependent CME (e.g., transferrin and epidermal growth factor receptor (EGFR)) is a well understood pathway [12,16]. Endocytosis by this pathway occurs through the invagination of plasma membrane regions having a high density of the cytosolic protein triskelion clathrin. Both receptor-dependent and independent CME results in eventual lysosomal sequestration, following movement of entrapped materials from early endosome to the late endosome, finally merging with lysosome to form the lysosome-endosome hybrid. Caveolin-mediated endocytosis (CvME), another mechanism of non-phagocytic endocytosis, occurs through a flask-shaped invagination of the plasma membrane [12]. As this pathway does not merge with lysosomes, pathogens have been shown to take advantage of this pathway to escape lysosomal enzymatic degradation [17,18]. Likewise, designing nanomedicines to target delivery of agents through CvME, thus avoiding degradation by the lysosomal enzymes, can be beneficial. For example, plasmid DNA delivery in KB cells (a sub-line of the cervical carcinoma HeLa cell line) by PEGylated, folate-targeted nanoparticles utilizes the CvME pathway [19].

Macropinocytosis and other routes of clathrin- and caveolae-independent endocytosis are less well understood pathways. Macropinocytosis involves the internalization of particles greater than 1 μm [12]. Macropinocytosis is an actin-dependent process resulting in internalization of particles via cell membrane “ruffling”. Endocytosis of particles by this pathway results in the formation of large endocytic vesicles called macropinosomes, which eventually merge with the lysosomal compartment. Cellular uptake of titanium dioxide nanoparticles in human prostate cancer cells has been shown to occur by macropinocytosis, in addition to clathrin- and caveolae-mediated endocytic routes [20].

### **Autophagy**

**Figure 2:**
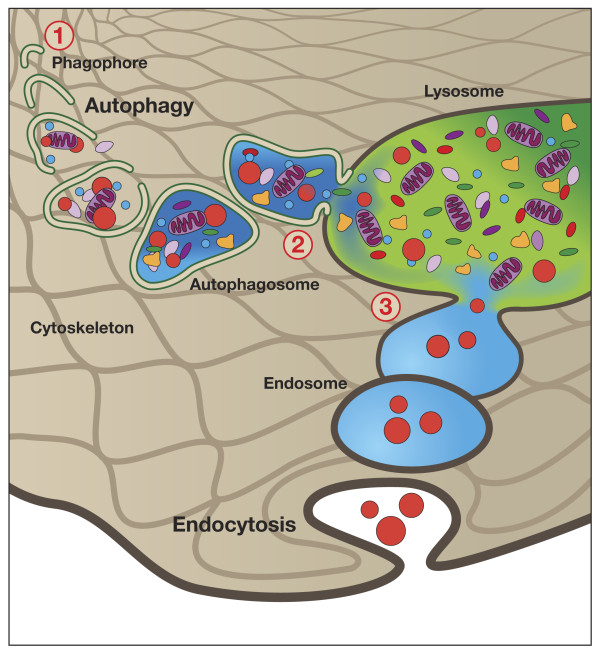
**Autophagy. 1)** During autophagy, a double layer membrane, the autophagosome, is formed that surrounds proteins and damaged organelles destined for degradation. **2)** The autophagosome then merges with the lysosome, where hydrolytic enzymes in the lysosome dismantle the autophagosome contents. **3)** The autophagy pathway is interconnected with the endocytosis pathways, with most endosomes eventually merging with the lysosome.

The macroautophagy pathway (defined in this review as “autophagy”) was first described by Christian De Duve in 1963 [21]. Autophagy (literally “self-eating”) involves the sequestration of cargo (e.g., cellular organelles, proteins, pathogens) in double-membrane structures called autophagosomes [3] (see Figure2). The fusion of autophagosomes with lysosomes, forming autolysosomes, results in the breakdown of encapsulated materials to components which are then available for cell growth and homeostasis. Recently, tremendous progress has been made in characterizing the autophagy protein machinery and signaling cascades, resulting in an explosion of applied research in autophagy [3]. As described in detail below, a growing body of literature suggests that intracellular nanoparticles may undergo autophagic sequestration, and autophagy dysfunction may play an important role in nanoparticle toxicity.

The autophagy pathway is evolutionarily conserved from yeast to mammals, with the identification of over 30 autophagy-related genes (ATG), and is regulated by several signaling cascades, e.g., mTOR and JNK [3,22]. Autophagy can be assessed by electron microscopy to observe autophagosomes and autolysosomes, by evaluation of the autophagy biomarker LC3-II, by use of lysosomotropic dyes, and by the use of genetically altered cells and animals, e.g., GFP-LC3 [23]. Activation of autophagy can occur in the event of stress due to starvation, depletion of growth factors, endoplasmic reticulum stress, oxidative stress and infection [22]. While autophagy is generally considered a nonselective response to these cellular stress conditions, autophagy is also reported to be homeostatic and selective in the removal of damaged organelles, ubiquitinated proteins, and pathogens [24]. For example, the autophagy adapter protein p62 binds to ubiquitinated protein aggregates and the autophagosome marker protein LC3, targeting these protein aggregates for degradation in the autolysosome [25,26]. There is also evidence that autophagy can selectively compartmentalize nanomaterials.

Nanomaterials have been visualized within the double membrane of autophagosomes following treatment of alveolar macrophages with carbon black nanoparticles, non-small cell lung cancer cells with EGFR-targeted gold-coated iron oxide nanoparticles, human mesenchymal stem cells with quantum dots, dendritic cells with alumina nanoparticles, and murine macrophages and human lung adenocarcinoma with silica nanoparticles [27-31]. However, as imaging techniques have been associated with artifacts, there is a need for quantitative methods to validate these findings [8,9,11]. A variety of nanoparticles have also been shown to induce dysfunction of the autophagy pathway [2,32], and this may be involved in their mechanism of toxicity. As will be discussed in subsequent sections, defects in the autophagy pathway have been associated with a number of pathologies in humans such as chronic infection, muscular disorders, cancer, and neurodegenerative disease [33].

### **Lysosomal dysfunction as a toxic mechanism**

**Figure 3:**
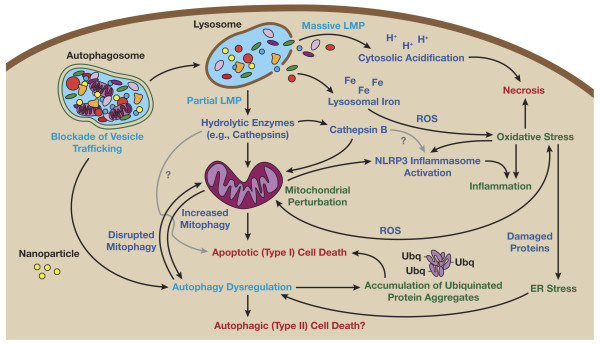
**Mechanisms of autophagy and lysosomal dysfunction toxicity.** The initiators of autophagy and lysosomal dysfunction toxicity, displayed in light blue text in the figure, include blockade of vesicle trafficking, lysosomal membrane permeabilization (LMP), and autophagy dysregulation. Nanoparticles could potentially cause autophagy dysfunction by overloading or directly damaging the lysosomal compartment, or altering the cell cytoskeleton, resulting in blockade of autophagosome-lysosome fusion. Nanoparticles could also directly affect lysosomal stability by inducing lysosomal oxidative stress, alkalization, osmotic swelling, or causing detergent-like disruption of the lysosomal membrane itself, resulting in LMP. Toxic effectors (lysosomal iron, cytosolic acidification, hydrolytic enzymes, reactive oxygen species, and the NLRP3 inflammasome) are displayed in dark blue. Conditions resulting from effector-mediated loss of homeostasis (oxidative stress, inflammation, ER stress, disrupted mitophagy, accumulation of ubiquitinated protein aggregates, and mitochondrial perturbation) are displayed in green. Finally, this loss of homeostasis can result in the cell death pathways necrosis, and Apoptotic (type I) and autophagic (type II) cell death; displayed in red (see text for details).

As will be discussed below, a great variety of nanomaterials have been associated with lysosomal dysfunction (see Table 1). One common form of lysosomal dysfunction that has been associated with nanomaterial treatment is lysosome membrane permeabilization (LMP). LMP is a recognized cell death mechanism that can result in mitochondrial outer membrane permeabilization through several mechanisms, including lysosomal-iron mediated oxidative stress, and release of cathepsins and other associated lysosomal hydrolases [34] (see Figure3). Mitochondrial permeabilization resulting from partial LMP can induce reactive oxygen species (ROS) generation and apoptosis, while massive LMP can cause cytosolic acidification and necrosis. Methods to detect LMP and lysosomal function include evaluation of the cellular distribution of pH-dependent lysosomotropic (acidotropic) dyes (e.g., acridine orange and neutral red), lysosomotropic particles (e.g., gold-coupled albumin and fluorescence-labeled dextran) or lysosomal proteins (e.g., immunostaining of lysosomal hydrolases) [34].

**Table 1:** A summary of nanomaterial–induced lysosomal perturbation

**Nanomaterial**	**Size and charge of the nanomaterial**	**Models**	**Experimental technique used to evaluate lysosomal perturbation,** **e.g. lysosomal membrane permeabilization (LMP)**	**Reference**
Multi-wall carbon nanotube	<8 nm, 20–30 nm, >50 nm*	3 T3 fibroblast; hT bronchial epithelial cells; RAW macrophages	Acridine orange staining (change from lysosomal red to cytosolic green fluorescence)	[35]
mercaptopropanoic acid-coated gold nanoparticles	5 nm; negative charge^#^	Mytilus edulis (blue mussel)	Neutral red retention assay in the haemolymph (loss of dye from the lysosomes to cytosol)	[36]
Titanium dioxide nanoparticles	<100 nm^#^	Rainbow trout gonadal tissue (RTG-2 cells)	Neutral red retention assay (loss of dye from the lysosomes to cytosol)	[37]
G5-PAMAM dendrimer	5 nm; positive charge^#^	KB cells, a sub-line of the human cervical carcinoma HeLa cell line	Measurement of lysosomal pH using dextran-fluorescein conjugate	[38]
Glass wool	3-7 μm*	Mytilus edulis (blue mussel)	Neutral red retention assay (loss of dye from the lysosomes to cytosol)	[39]
Titanium dioxide nanoparticles	5 nm; neutral^#^	L929 mouse fibroblast	Transmission electron microscopy (TEM)	[40]
Silver nanoparticles	25 nm; negative charge^#^	Crassostrea virginica (Oyster)	Neutral red retention assay (loss of dye from the lysosomes to cytosol)	[41]
Fullerene (C60) nanoparticles	~150 nm^#^	Crassostrea virginica (Oyster)	Neutral red retention assay (loss of dye from the lysosomes to cytosol)	[42]
Silica particles	Micron scale*	Mouse peritoneal macrophages	Acridine orange staining (change from lysosomal red to cytosolic green fluorescence) and release of lysosomal enzymes (Acid phosphatase and β-glucuronidase activity)	[43]
TNF-bp20-K PEG monomer (38 kDa)	nanoscale*	Sprague–Dawley rats (Renal cortical tubular epithelium)	Histopathology evaluation (vacuolization)	[44]
Titanium dioxide nanoparticles	15 nm*, 461 nm (PBS); negative charge^#^	16HBE14o-cells, human bronchial epithelial cells	Acridine orange staining (change from lysosomal red to cytosolic green fluorescence), Immunostaining for cytosolic cathepsin B	[45]
Polyalkyl-sulfonated C60	nanoscale*	Sprague–Dawley rats (liver and kidney)	Histopathology, TEM	[46]
Zinc oxide nanoparticles	10 nm*, 229 nm in PBS + 5 % mouse serum^#^; negative charge (in PBS + 5 % mouse serum)^#^	THP-1 cells, human monocytic cell line	Acridine orange staining (change from lysosomal red to cytosolic green fluorescence)	[47]
Titanium dioxide nanoparticles	Nanospheres 60–200 nm, Long nanobelts 15–30 μm, Short nanobelts 0.8-4 μm; slightly negative^#^	C57BL/6 alveolar macrophages	TEM, cytosolic cathepsin B, Acridine orange staining (change from lysosomal red to cytosolic green fluorescence)	[48]
Polystyrene nanoparticles	110 nm; positive charge^#^	Human macrophages	Cytosolic cathepsin B, Acridine orange staining (change from lysosomal red to cytosolic green fluorescence)	[49]

*characterized by manufacturer; ^#^characterized by author.

As lysosomal dysfunction has been implicated in disease pathogenesis, the association of nanoparticle exposure and lysosomal dysfunction may have relevance to nanomaterial-induced toxicities, especially chronic toxicities. The lysosomal degradation pathways play a vital role in cellular homeostasis, and lysosomal dysfunction has been associated with several disease states, termed lysosomal storage disorders [33,50]. Lysosomal storage disorders, resulting from the lack of functional lysosomal enzymes or other crucial component proteins, such as H^+^-ATPase, manifest as degenerative diseases of the nervous and musculoskeletal systems, such as mucopolysaccharidoses and sphingolipidoses [51,52]. Lysosomal dysfunction resulting in an accumulation of unmetabolized substrates in the lysosome and lysosomal overload can have several possible deleterious consequences to the cell, including LMP and defects in intracellular trafficking, as well as altered intracellular signaling and gene expression [50]. Many lysosomal diseases also show evidence of autophagy dysfunction, with blockade of autophagosome and lysosome fusion, and accumulation of autophagosomes and autophagy substrates (e.g., ubiquitinated protein aggregates) [53]. Autophagy dysfunction can result from lysosomal overload or alkalization, which prevents autophagosome-lysosome fusion. Similar to lysosomal dysfunction, dysfunction of the autophagy pathway itself has also been linked to a variety of diseases [33].

### **Lysosomal dysfunction by nanomaterials**

Lysosomotropic agents, including particles, have been known for several decades to cause lysosomal dysfunction and associated toxicities of lung, liver and kidney [4,54]. Consequently, lysosomal dysfunction may be a common outcome of nanoparticle exposure since, as described above, nanoparticles are commonly sequestered within the lysosomal compartment. Additionally, nanoparticles have properties of substances known to cause lysosomal disorders [54,55], including enzyme inhibiting ability [56,57] and biopersistence. Correspondingly, many researchers have observed nanomaterial-induced lysosomal dysfunction (see Table 1). LMP has been identified as a potential mechanism of carbon nanotube toxicity in human fibroblasts [35] and cationic polystyrene nanosphere toxicity in human macrophages [58], and in both cases LMP was associated with loss of mitochondrial membrane potential and apoptosis. In the case of the cationic polystyrene nanosphere, a “proton sponge” theory has been proposed to explain lysosomal rupture, in which the high proton buffering capacity of the particle surface amines results in excessive proton pump activity and osmotic swelling [58]. Other cationic nanoparticles, such as cationic polyamidoamine (PAMAM) dendrimers, have also been shown to induce LMP, as well as loss of mitochondrial membrane potential and apoptosis, with a similar proton sponge mechanism proposed [38].

LMP has also been noted in mussels treated with nanoscale glass wool and gold nanoparticles [36,39], as well as oysters treated with fullerene and silver nanoparticles [41,42]. In these cases, nanomaterials concentrated within the lysosomal compartment of the hepatopancreas, and lysosomal destabilization was identified by evaluating cytosolic accumulation of lysosomotropic dyes or lysosomal enzymes. Lysosomal destabilization has also been proposed as a mechanism of nanoscale titanium dioxide cytotoxicity in mouse fibroblast cells [40] and human bronchial epithelial cells [45]. Lung injury induced by zinc nanoparticles in mice was also attributed to lysosomal destabilization following macrophage uptake and pH-dependent zinc ion dissolution [47]. This effect on lysosomal stability does not appear to be unique to nanoscale particles, as previous studies have implicated changes in lysosomal permeability and the subsequent release of lysosomal enzymes as one of the mechanisms involved in the induction of apoptosis in alveolar macrophages by silica microparticles [59]. However, there are comparative studies of nano- versus micro-scale gold nanoparticles that have suggested nanoscale particles have a much greater potency for induction of LMP [36]. Recently, inflammatory responses to particles and fibers, including silica, asbestos, high aspect ratio nanoscale titanium dioxide fibers, carbon nanotubes and amino-functionalized polystyrene nanoparticles, have been proposed to result from cathepsin B-mediated activation of the NLRP3 inflammasome following LMP [48,49,60,61]. Thus, in addition to providing an indirect mechanism for nanoparticle-induced ROS generation and oxidative stress through lysosomal iron release or mitochondrial damage, lysosomal dysfunction also provides a mechanism for nanoparticle-mediated inflammation (see Figure3).

### **Autophagy dysfunction as a toxic mechanism**

Autophagy dysfunction is defined as excessive autophagy induction or blockade of autophagy flux. Autophagy dysfunction is recognized as a potential mechanism of cell death, resulting in either apoptosis or autophagic cell death (also referred to as type II programmed cell death) [34] (see Figure3). However, since the majority of data supports autophagy as a pro-survival pathway rather than a cell death pathway, with evidence for a direct role in cell death only coming from artificial systems in which apoptosis is chemically or genetically suppressed, the role of autophagy in “programmed cell death” is debatable [34]. Several mechanisms have been proposed to explain autophagy dysfunction-mediated apoptosis. Since agents that block autophagy flux, such as the proton pump inhibitor bafilomycin A1, also commonly predispose cells to LMP, apoptosis may in some cases be the result of LMP release of pro-apoptosis mediators such as cathepsins [62]. Recently, evidence has also been presented suggesting the autophagy proteins LC3B and ATG5 may directly activate caspase-dependent cell death through interactions with Fas and Fas-associated protein with death domain (FADD), respectively [63,64]. Additionally, a calpain truncated form of ATG5 has been shown to compete with the apoptosis suppressor protein Bcl-X_L_, and may thereby activate apoptosis [65]. JNK-mediated phosphorylation of BCL-2 may also be a common pathway for autophagy and apoptosis regulation. There is evidence that low levels of BCL-2 phosphorylation by JNK results in release of the pro-autophagy protein Beclin-1, and upon further phosphorylation there is eventual release of the pro-apoptotic factors BAX and BAK [66]. Thus, excessive JNK activation, as has been shown to occur in autophagy blockade [67], might result in release of pro-apoptosis factors through BCL-2 phosphorylation. As discussed in the following sections, autophagy flux blockade has also been associated with accumulation of ubiquitinated proteins and mitochondrial dysfunction, providing additional mechanisms for autophagy-induced apoptosis.

Similar to lysosomal dysfunction, dysfunction of the autophagy pathway has also been linked to a variety of diseases [33]. There is evidence that autophagy dysfunction plays a role in both cancer development and progression [68]. Evidence suggests that defective autophagy can lead to cancer development, possibly by allowing the accumulation of damaged organelles, such as mitochondria, that can then induce oxidative stress, inflammation and DNA damage. For example, one of the alleles of the pro-autophagy gene Beclin-1 is commonly deleted in several forms of cancer, including breast, ovarian and prostate, suggesting a tumor suppressor function for the autophagy pathway. In agreement, the monoallelic knock-out of Beclin-1 in mice enhances susceptibility to cancer [68,69]. There is also data to suggest that once established, autophagy may play a pro-survival role, allowing tumors to grow under nutrient deprived conditions or survive chemotherapy-induced stress. Indeed, autophagy disruptors, such as the antimalarial drug chloroquine, have been shown to synergize with chemotherapeutic agents in killing cancer cells [70]. As discussed below, some nanoparticles have also recently been shown to potentiate the cytotoxicity of chemotherapeutics by disrupting autophagy.

Defective autophagy has also been associated with Crohn’s disease, a chronic inflammatory disease of the intestine, as well as with neurodegenerative conditions, such as Parkinson’s and Alzheimer’s disease, and may play a role in disease development [33,71-73]. Mutations in the autophagy-related genes ATG16L/IRGM and PINK1/Parkin have been linked to Crohn’s and Parkinson’s diseases, respectively [33,71,74]. In the case of Crohn’s disease, disruption of autophagy’s role in immunity and inflammation may be involved in disease development, while for neurodegenerative diseases such as Parkinson’s and Alzheimer’s disease, blockade of autophagy-mediated elimination of disease-associated proteins (e.g., amyloid beta and alpha synuclein) or damaged mitochondria may be involved [33,75-78]. Disruption in autophagosome trafficking to the lysosome has also been implicated in neurodegenerative diseases, such as mutant dynactin associated motor neuron disease and amyotrophic lateral sclerosis, as well as myopathies, such as inclusion body myopathy [33]. As exposure to airborne pollution has been associated with Alzheimer and Parkinson-like pathologies, and nanoparticles are the primary particle number and surface area component of pollution-derived particulates, we (S.T.S.) have recently postulated a relationship between nanoparticle-induced autophagy dysfunction and pollution-associated neurodegeneration [2]. In support of this hypothesis, autophagy dysfunction has recently been postulated as a mechanism of manganese nanoparticle-induced cytotoxicity in dopaminergic neuronal cells [79].

### **Autophagy induction by nanomaterials**

**Figure 4:**
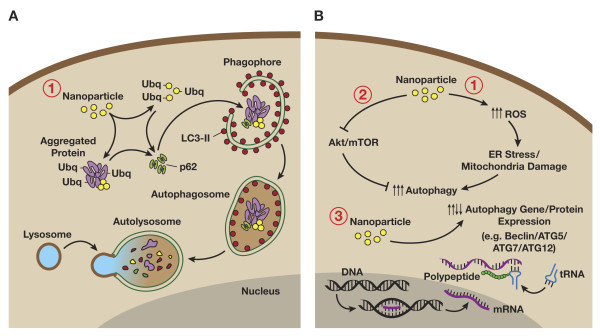
**Nanoparticle-induced autophagy. A)** Recent evidence supporting ubiquitination of nanomaterials directly, or indirectly through colocalization with protein aggregates, suggests that cells may select nanomaterials for autophagy through a p62-LC3 II pathway similar to invading pathogens (see text). **B)** Data also supports nanomaterial-induced alteration of autophagy signaling pathways, including: **1)** induction of oxidative stress-dependent signaling (e.g., ER stress, mitochondrial damage), **2)** suppression of Akt-mTOR signaling, and **3)** alteration of autophagy related gene/protein expression.

Autophagy perturbation, both induction and blockade, has been reported consistently across several classes of nanomaterials and biological models (Table 2). There are several plausible mechanisms of autophagy induction by nanomaterials (Figure4). Nanomaterials may induce autophagy through an oxidative stress mechanism [1], such as accumulation of oxidatively damaged proteins and subsequent endoplasmic reticulum stress, or mitochondrial damage [22]. The involvement of oxidative stress in induction of autophagy by nanomaterials is supported by a study in which fullerene-induced autophagy in HeLa cells was dependent upon photoactivation and suppressed by the antioxidants N-acetyl-L-cysteine, reduced glutathione, and L-ascorbic acid [96]. Alternatively, nanomaterials may directly affect autophagy dependent signaling pathways or gene/protein expression (see Table 2). There also remains the likely possibility that autophagy induction by nanomaterials may simply be an attempt to degrade what is perceived by the cell as foreign or aberrant, similar to bacteria and other pathogens. As discussed above, nanoparticles are commonly observed within the autophagosome compartment, suggesting that activation of autophagy is a targeted attempt to sequester and degrade these materials following entrance into the cytoplasm. Cytoplasmic bacteria undergo ubiquitination, as well as colocalize with polyubiquitin complexes, and are then targeted to the autophagosome by p62 [99,100]. Recent evidence also supports ubiquitination of nanomaterials directly, or indirectly through colocalization with ubiquitinated protein aggregates, suggesting that cells may indeed select nanomaterials for autophagy through a pathway similar to invading pathogens [28,101-105].

**Table 2:** A summary of nanomaterial-induced autophagy perturbation

**Nanomaterial**	**Size and charge of the nanomaterial**	**Models**	**Autophagy markers examined**	**Experimental techniques used to evaluate autophagy perturbation**	**Reference**
Manganese nanoparticles	30 - 50 nm*	Rat N27 dopaminergic neuronal cells	LC3 and Beclin 1	Immunoblot, GFP- LC3 transfection	[79]
Neodymium oxide nanoparticles	80 nm*	NCI-H460 human lung cancer cells	None	TEM, acridine orange staining	[80]
C60 fullerene pentoxifylline dyad nanoparticles	79 nm^#^	Mouse neuroblast neuro 2A cells	LC3	Immunoblot and TEM	[81]
Fullerenol	20 nm; negative charge^#^	LLC-PK1 porcine kidney cells	LC3	Lysotracker assay, Immunoblot, TEM	[32]
Gold nanoparticles	10, 20, 50 nm; negative charge^#^	Rat kidney (NRK) cells	LC3	Immunoblot, GFP- LC3 transfection, TEM	[82]
Iron oxide nanoparticles	115 nm; negative charge^#^	A549 human lung cancer cells	LC3, ATG5, ATG12; AKT signaling	Immunoblot	[83]
Polymeric nanoparticles (Eudragit RS)	54 nm; positive charge^#^	NR8383 rat alveolar macrophage cell line	LC3	Immunoblot, TEM, LC3-Immunostaining	[84]
EGFR-plasmonic magnetic nanoparticles	73 nm; negative charge^#^	Non-small cell lung cancer cells	LC3	Immunoblot, TEM, GFP-LC3 transfection	[31]
Yttrium oxide nanoparticles	177 nm^#^	HeLa cells	LC3	Immunoblot, TEM, GFP-LC3 transfection	[85]
Ytterbium oxide nanoparticles	279 nm^#^				
Fullerene C60 nanoparticles	50-100 nm; negative charge^#^	MCF-7 human breast cancer cell line	LC3	Immunoblot, GFP- LC3 transfection	[86]
Uncoated, ultrasmall superparamagnetic iron oxide (USPIO) nanoparticles	8 nm; positive charge*	HCEC Human brain endothelial cells	LC3	Immunoblot, TEM	[87]
Titanium dioxide nanoparticles	21 nm; negative charge*				
Silica nanoparticles	25, 50 nm; negative charge*				
Cadmium selenide quantum dot	5.1 nm^#^	LLC-PK1 porcine kidney cells	LC3	Immunoblot, TEM, Lysotracker assay	[88]
Indium gallium phosphide quantum dot	3.7 nm^#^				
PAMAN Dendrimer	Several different generations (varying in sizes and charge)*	A549 human lung cancer cells; Balb\c mice	LC3, AKT signaling	Immunoblot, TEM, GFP-LC3 transfection	[89]
Silica nanoparticles (spheres, worms, cylinders)	Several^#^	A549 human lung cancer cells, RAW 264.7 mouse macrophages	LC3	Immunoblot, TEM	[27]
Fullerene C60/70 nanoparticles	100 nm^#^	Rat C6 glioma cell line	None	Acridine orange staining	[90]
Iron core with gold shell nanoparticles	10 nm^#^	OEMC1 human oral cancer cell line	LC3	Immunoblot, TEM, LC3 Immunostaining	[91]
Titanium dioxide nanoparticles	<25 nm*	HT29 human colon cancer cell line	None	GFP-LC3 transfection	[92]
Palladium nanoparticles	5-10 nm	Peripheral blood mononuclear cells (PBMC)	LC3	TEM	[93]
Single walled carbon nanotube- carboxylic acid	nanoscale*	A549 human lung cancer cell line/Balb/c mice	LC3, AKT signaling	Immunoblot, TEM, ATG6 siRNA transfection	[94]
Gold nanoparticles	22 nm; negative charge^#^	MRC-5 human lung fibroblast cells	LC3, ATG7	Immunoblot, TEM	[95]
Fullerene C60 nanocrystals	20-100 nm^#^	MCF-7 human breast cancer cell line, HeLa human cervical cancer cell line	LC3	Immunoblot, TEM, GFP-LC3 transfection	[96]
Samarium oxide; Europium oxide; Gadolinium oxide; Terbium oxide nanoparticles	50 nm^#^	HeLa human cervical cancer cell line	LC3	Immunoblot, TEM, GFP-LC3 transfection	[97]
Fullerenol nanoparticles	7.1 nm^#^	HUVEC human umbilical vein endothelial cell line	LC3	Immunoblot, TEM	[98]
Quantum dots	nanoscale*	Human mesenchymal stem cells	LC3	LC3 immunostaining, TEM	[30]
Alpha alumina nanoparticles	60 nm^#^	Dendritic cells	LC3	LC3 immunostaining, Immunoblot, TEM	[28]

*characterized by manufacturer; ^#^characterized by author.

**Figure 5:**
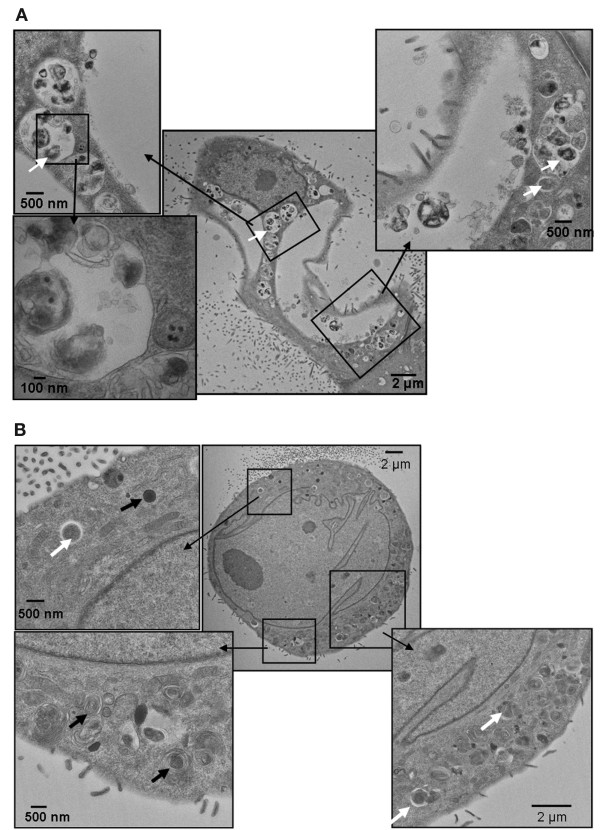
**Transmission electron micrographs detailing autophagic vacuoles.** Porcine kidney cells (LLC-PK1) were treated for 24 h with either **(A)** 10 nM CdSe or **(B)** 100 nM InGaP quantum dots. The white arrows indicate autophagic vacuoles containing cellular debris. The black arrows indicate lysosomal remnants, consisting of multilamellar vacuoles and electron-dense deposits. (Stern ST, et al, Induction of Autophagy in Porcine Kidney Cells by Quantum Dots: A Common Cellular Response to Nanomaterials? Tox. Sci. 2008, 106(1), 140–152, by permission of Oxford University Press.)

Nanoscale neodymium oxide potently induces autophagy, as assessed by electron microscopy, in non-small cell lung cancer NCI-H460 cells [80]. Induction of autophagy has also been observed following treatment with several other rare earth oxide nanocrystals, such as gadolinium oxide, in HeLa cells [97]. Induction of autophagy in human fibroblasts by gold nanoparticles, currently in development for drug delivery applications [106], was associated with upregulation of autophagy proteins LC3 and ATG7 [95]. Quantum dots, currently under development for a broad range of biomedical imaging applications, have also been shown to induce autophagy in a variety of cell lines, including porcine kidney cells (Figure5) and human mesenchymal stem cells [30,88]. As the underlying composition (e.g., CdSe, InGaP) and surface chemistries (e.g., protein coated, silica coated, PEGylated) of the quantum dots used in these studies varied substantially, it would suggest that the commonality of nanoscale size was a significant factor in eliciting this common autophagic response. Consistent with this assumption, autophagy was not induced by quantum dots that had a tendency to aggregate to micro-scale particles intracellularly [30]. Nanoscale size dependence was also noted for neodymium oxide nanoparticle autophagy-induction [80], with larger particles having reduced activity.

Recently, it has been reported that cationic PAMAM dendrimers and carboxylated carbon nanotubes could induce autophagosome accumulation in human lung adenocarcinoma cells [89,94]. Cytotoxicity associated with dendrimer or carbon nanotube treatment of the lung cancer cells was blocked by cotreatment with the autophagy inhibitor 3-methyladenine (3-MA) or knockdown of the autophagy gene ATG6, suggesting involvement of the autophagy pathway in cytotoxicity. Data further suggested that autophagy induction by both the cationic dendrimer and carbon nanotube was the result of inhibition of mTOR signaling. However, the possibility of autophagy flux blockade in concert with autophagy induction through the mTOR pathway was not evaluated. Treatment of mice with cationic PAMAM dendrimers or carbon nanotubes by intratracheal instillation resulted in lung inflammation, as determined by lung wet/dry ratios, immune cell infiltration, and histology. Consistent with the in vitro findings, autophagosome accumulation was also observed in the lung tissue of mice treated with the carboxylated carbon nanotubes. Also consistent with the in vitro findings, cationic dendrimer and carbon nanotube-induced lung inflammation, and associated animal death, could be prevented by cotreatment with 3-MA. In further support of cationic PAMAM dendrimer interaction with the autophagy pathway, liver lesions observed in mice treated intraperitoneally (i.p.) with cationic PAMAM dendrimers in a separate study had histological features typical of lysosomal disorders observed with polycationic drugs, including vacuolization [54,107,108].

### **Autophagy blockade by nanomaterials**

In the majority of the studies (Table 2), autophagosome accumulation induced by nanomaterial treatment was associated with cell death. As mentioned above, there is scant evidence of autophagy as an actual effector of cell death, and cytotoxicity resulting from blockade of autophagy’s pro-survival mechanisms would appear the more likely scenario. Since blockade of autophagy flux and autophagy induction can both lead to autophagosome accumulation [23], and the possibility of autophagy blockade was most often not investigated, the mechanism of nanomaterial-induced autophagy accumulation in many cases is uncertain. In cases where autophagy flux has been evaluated, disrupted or blocked autophagy flux is more often seen upon exposure to nanomaterials. For example, fullerene, the fullerene derivative fullerenol, and gold nanoparticles have been shown to block autophagy flux in various cell lines [32,82,86]. In renal proximal tubule cells, blockade of autophagy flux by fullerenol, as measured by increased autophagosome marker LC3-II and autophagy flux marker p62, was associated with mitochondrial depolarization and disruption of the actin cytoskeleton [32]. In support of the in vivo relevance of these findings, an acute toxicity study of a water soluble polyalkylsulfonated fullerene in rats identified a lysosomal overload nephropathy that is consistent with blockade of lysosomal trafficking [46].

There is a substantial body of literature linking nanomaterial-induced autophagy dysfunction with mitochondrial damage [27,32,79,83,84,91]. Disruption of the autophagy pathway by genetic knockout has also been associated with accumulation of dysfunctional mitochondria, as well as accumulation of ROS, thus providing a potential link between nanomaterial-induced autophagy blockade and oxidative stress [109]. Nanomaterial-induced autophagy blockade may also be a mechanism of nanomaterial-associated inflammation, as there is evidence that autophagy plays an important role in negatively regulating the NLRP3 inflammasome [110]. Autophagy blockade may result in mitochondrial dysfunction by preventing removal of damaged mitochondria that are normally degraded through the autophagy pathway [111]. Postulated to be a mechanism to eliminate damaged mitochondria, there is also evidence that mitochondrial depolarization actually precedes autophagy induction [112]. Thus, it is conceivable that a combination of autophagy induction and autophagy blockade, as might be expected with nanoparticles, could result in an increased number of depolarized dysfunctional mitochondria that cannot be cleared as a result of the impaired autophagy flux. Indeed, the combination of autophagy induction, by Akt inhibition, and autophagy flux blockade, by chloroquine treatment, results in accumulation of abnormal, depolarized mitochondria and ROS in human prostate cancer cells [113]. Similarly, simultaneous starvation-induced autophagy and autophagy flux blockade, by treatment with the proton pump inhibitor bafilomycin A1, also results in mitochondrial depolarization in cervical cancer cells [114].

The relationship between nanomaterial-induced autophagy blockade and mitochondrial is intriguing [32], as blockade of autophagy flux with concomitant mitochondrial dysfunction has also been observed in alveolar macrophages from smokers [29]. Mitochondrial dysfunction in smokers’ alveolar macrophages could be reproduced by treatment of alveolar macrophages from non-smokers with bafilomycin A1, a proton pump inhibitor that is also known to block autophagy flux by inhibition of the lysosomal proton pump [29]. Autophagy blockade and mitochondrial dysfunction in smokers’ macrophages were also reproduced by treatment of alveolar macrophages from non-smokers with cigarette smoke extract or, most importantly, nanoscale carbon black, a component of cigarette smoke, thus implicating nanoscale particulates in this process [29]. This is not surprising, as studies have observed autophagosome accumulation in human umbilical vein endothelial cells treated with nanoscale carbon black [115]. Recently, cytotoxicity associated with autophagosome accumulation and mitochondrial damage was also observed following treatment of rat alveolar macrophages with Eudragit® RS nanoparticles [84].

The autophagy blockade observed in smokers’ alveolar macrophages was associated with reduced pathogen phagocytosis and reduced delivery of phagocytosed pathogens to the lysosome, which might explain the increased susceptibility of smokers to bacterial infection [29]. Previous studies have shown nanoscale particulates to have a greater potency than larger particles of the same material in retarding lung particle clearance, resulting in lung particle overload [116]. This could conceivably be in part due to a greater blockade of the autophagy pathway by the nanoscale particles, as clearance of pathogens and other immune functions, including regulation of proinflammatory responses, have been attributed to the autophagy pathway [117].

Another interesting phenomenon associated with autophagy blockade in the alveolar macrophage from smokers was accumulation of polyubiquitinated protein complexes [29]. This accumulation of ubiquitinated proteins has also been observed previously in human vascular endothelial cells treated with fullerenes, and was also associated with autophagy dysfunction [98]. The disruption in clearance of polyubiquitinated proteins upon autophagy dysfunction is further evidence that autophagy, in addition to the proteasome, is involved in degradation of ubiquitin-targeted proteins [118].

### **Mechanisms of lysosomal dysfunction and autophagy blockade by nanomaterials**

There are many plausible explanations for nanoparticle-induced lysosomal dysfunction (Figure3). In addition to the “proton sponge” hypothesis of LMP for cationic nanoparticles described above, involving osmotic swelling and membrane rupture, another direct mechanism that might account for nanoparticle-induced LMP is the generation of ROS, as studies have shown that reactive oxygen species can induce LMP in cells in culture [119]. This may be a likely mechanism, as many nanoparticles can induce ROS, and the oxidative stress paradigm of nanoparticle-induced toxicity is by far the most accepted [1]. Agents that alkalinize the lysosome, such as the proton pump inhibitor bafilomycin A1, also commonly predispose cells to LMP [62]. Supportive of an alkalization mechanism, gold nanoparticles have recently been shown to increase lysosomal pH in rat kidney cells, causing lysosomal dysfunction [82]. Disruption of lysosomal trafficking has also been shown to increase LMP susceptibility. For example, agents that block lysosomal trafficking by disruption of the cytoskeleton, such as vinca alkaloids, also induce LMP and cathepsin-dependent apoptosis [120]. Likewise, mutation of the LYST gene that encodes a lysosomal trafficking regulator, seen in Chediak-Higashi Syndrome, results in dysfunctional lysosomes with a greater propensity toward LMP [121]. Recently, our laboratory found that fullerenol treatment of kidney cells resulted in blockade of autophagy flux, potentially by disruption of actin cytoskeleton-mediated lysosome trafficking [32].

Disruption of lysosomal trafficking is a principle mechanism for blockade of autophagy flux, resulting in accumulation of autophagic and lysosomal vacuoles. There are several plausible mechanisms by which nanoscale particulates might disrupt autophagy and lysosomal trafficking (Figure3). Lysosomal overload by particulates has been proposed as a mechanism for blockade of autophagy flux by cigarette smoke in alveolar macrophages [29]. The overload of alveolar macrophage lysosomes with indigestible material, such as smoke particulates and asbestos nanofibers, has been shown to disrupt lysosomal fusion with other cellular compartments, resulting in vacuole accumulation [122]. This would suggest that biopersistent nanomaterials are of greater concern. Indeed, biopersistent gold nanoparticles have been shown to accumulate in the lysosome, blocking autophagosome-lysosome fusion fusion and lysosomal degradation [82]. This association between biopersistent materials and lysosomal dysfunction, presenting as intracellular vacuolation, has been observed previously in the kidneys of Sprague Dawley rats treated with polyethylene-glycol conjugated proteins, and in the liver of humans receiving polyvinyl pyrrolidone as a plasma expander [44,123].

Another likely mechanism of autophagy blockade is disruption of the cytoskeleton upon which the autophagy andlysosomal compartments rely for cellular trafficking. Agents that disrupt the microtubular cytoskeleton, such as vinblastine, cause blockade of autophagy flux during both selective, basal autophagy and nonselective, starvation-induced autophagy by preventing autophagosome-lysosome fusion [124-127]. By contrast, the actin cytoskeleton appears to be specifically involved in selective, quality control autophagy in both yeast [128,129] and mammalian cells [130]. During quality control autophagy in mammalian cells, which is responsible for removal of protein aggregates and damaged mitochondria, the ubiquitin binding deacetylase, histone deacetylase-6, has recently been shown to regulate autophagosome-lysosome fusion by actin remodeling [130]. Furthermore, latrunculin A, an agent that disrupts actin polymerization, blocks autophagosome-lysosome fusion during selective, basal autophagy [130]. As the studies described above detailing nanomaterial-induced autophagy blockade appear associated with disruption in mitochondrial and protein quality control autophagy, the actin cytoskeleton represents a plausible nanomaterial target.

A survey of protein binding of cellular extracts to silicon dioxide, titanium dioxide, and polystyrene nanoparticles and microparticles, with varying surface chemistries, found actin to be one of the most commonly bound proteins [131]. A similar actin binding tendency has been observed in our laboratory upon incubation of gold nanoparticles with human serum (unpublished data), even though actin is of low abundance in serum. In addition, our laboratory also has preliminary data that certain nanomaterials may alter actin polymerization (unpublished data). In further support of actin as a potential target of nanomaterial-induced autophagy dysfunction, cationic dendrimers have been shown to bind actin and inhibit actin polymerization in vitro [132], and induce autophagy dysfunction both in vitro and in vivo [89]. Treatment of human aortic endothelial cells and primary human dermal fibroblasts with carbon nanotubes [133] and gold nanoparticles [134], respectively, also results in disruption of the actin cytoskeleton, and both materials have also been shown to induce autophagy dysfunction in separate studies [94,95]. Additionally, researchers have shown that treatment of human umbilical vein endothelial cells with nanoscale iron oxide particles results in disruption of the actin and microtubule cytoskeletons, and coincident autophagy dysfunction [135].

### **Autophagy and lysosomal dysfunction as a therapeutic mechanism**

The interaction of nanomaterials with the autophagy and lysosomal pathways, and resulting dysfunction, is not necessarily always a disadvantageous scenario. As described above, LMP has been identified as a potential mechanism of cationic dendrimer cytotoxicity, but this LMP induction may also have benefits for drug delivery. Dendrimers are currently under development for a variety of drug delivery and imaging applications [136], and cationic dendrimer-induced LMP, as well as that of other cationic nanoparticles, has been used to enable lysosomal escape for cellular delivery of drugs, including gene therapies [137,138].

Many novel nanomedicine therapeutics are also under development utilizing autophagy dysfunction as a therapeutic mechanism. For example, a novel therapeutic vaccine is presently under development that utilizes alumina nanoparticles to transport bound antigen to autophagosomes of dendritic cells in an apparent p62-mediated fashion, block lysosomal degradation, and elicit a potent T-cell anti-tumor response [28]. Iron oxide and iron-coated gold nanoparticles currently being developed as potential therapeutics have been shown to selectively kill lung epithelial and oral squamous carcinoma cells, respectively, by an apparent autophagy-dependent mechanism [83,91].

As mentioned previously, autophagy disrupters are currently being evaluated in clinical trials for their ability to synergize with existing cancer therapies [70]. In order to identify new autophagy modulating agents for use as therapeutics, as well as evaluate responses to these agents, there is a need for methods to monitor autophagy. Since nanomaterials have been shown to interact with the autophagy pathway, they have been proposed as new tools to monitor autophagy [139]. An example of the use of a nanoparticle-based autophagy monitoring system has been described by Choi *et al*[140]. In this autophagy flux monitoring system, a fluorescent-peptide construct on the surface of a lysosomotropic polymeric nanoparticle is cleaved to a fluorescent form by the autophagy flux-dependent protease Atg4.

Recently, Lee *et al.* have demonstrated the ability of a water-dispersed neodymium fullerene derivative to synergize with doxorubicin to kill drug resistant MCF-7 human breast cancer cells [86]. The mechanism behind this synergy was shown to be dependent upon autophagy, as fullerene treatment resulted in dramatic autophagosome accumulation and the autophagy inhibitor 3-methyladenine blocked both autophagosome accumulation and drug synergy. However, treatment with the autophagy inducer rapamycin, an inhibitor of mTOR, had the opposite effect, actually antagonizing doxorubicin cytotoxicity. The authors concluded that blockade of autophagy flux and resulting futile autophagy, possibly due to particle overload and disruption of autophagosome-lysosome fusion, was the mechanism underlying the observed synergy. Fullerene interaction with the autophagy pathway is also currently being explored for the amelioration of amyloid-beta toxicity and treatment of Alzheimer’s disease [81]. Obviously, there is a need to understand the balance between the potential benefits of lysosomal dysfunctions for therapeutic purposes and potential harmful effects to the cell.

## **Conclusion**

The continued expansion of the field of nanotechnology requires a thorough understanding of the potential mechanisms of nanomaterial toxicity for proper safety assessment and identification of exposure biomarkers. With increasing research into nanomaterial safety, details on the biological effects of nanomaterials have begun to emerge. Researchers should be aware that nanomaterials can have detrimental effects on the autophagy and lysosomal pathways, resulting in toxicological consequences. Overall, expanding knowledge of the implications and biological significance of autophagy and lysosomal dysfunction has tremendous potential to aid in our understanding of nanotechnology risks, and design of safer nanomaterials and nanomedicines.

## **Competing interests**

The authors have no competing interests.

## **Authors’ contributions**

S.T.S. and P.P.A. performed literature reviews and wrote manuscript. R.M.C. provided critical review and assisted with manuscript generation. All authors read and approved the final manuscript
